# Fatty Acid Desaturases, Polyunsaturated Fatty Acid Regulation, and Biotechnological Advances

**DOI:** 10.3390/nu8010023

**Published:** 2016-01-04

**Authors:** Je Min Lee, Hyungjae Lee, SeokBeom Kang, Woo Jung Park

**Affiliations:** 1 Department of Horticultural Science, Kyungpook National University, Daegu 41566, Korea; jemin@knu.ac.kr; 2 Department of Food Engineering, Dankook University, Cheonan, Chungnam 31116, Korea; lee252@dankook.ac.kr; 3 Citrus Research Station, National Institute of Horticultural & Herbal Science, RDA, Seogwipo 63607, Korea; hortkang@korea.kr; 4 Department of Marine Food Science and Technology, Gangneung-Wonju National University, Gangneung, Gangwon 25457, Korea

**Keywords:** polyunsaturated fatty acid, fatty acid desaturase, transgenic system, health and development

## Abstract

Polyunsaturated fatty acids (PUFAs) are considered to be critical nutrients to regulate human health and development, and numerous fatty acid desaturases play key roles in synthesizing PUFAs. Given the lack of delta-12 and -15 desaturases and the low levels of conversion to PUFAs, humans must consume some omega-3 and omega-6 fatty acids in their diet. Many studies on fatty acid desaturases as well as PUFAs have shown that fatty acid desaturase genes are closely related to different human physiological conditions. Since the first front-end desaturases from cyanobacteria were cloned, numerous desaturase genes have been identified and animals and plants have been genetically engineered to produce PUFAs such as eicosapentaenoic acid and docosahexaenoic acid. Recently, a biotechnological approach has been used to develop clinical treatments for human physiological conditions, including cancers and neurogenetic disorders. Thus, understanding the functions and regulation of PUFAs associated with human health and development by using biotechnology may facilitate the engineering of more advanced PUFA production and provide new insights into the complexity of fatty acid metabolism.

## 1. Introduction

Polyunsaturated fatty acids (PUFAs) consist of more than two double bonds and are a group of critical nutrients that modulate brain development and cognition as well as many diseases such as cardiovascular disease, cancers, and diabetes [1,2]. Twenty-carbon PUFAs are precursors of eicosanoids that regulate inflammatory and immune responses through pro- and anti-inflammatory activities; docosahexaenoic acid (DHA, 22:6*n*-3) is a precursor of anti-inflammatory docosanoids [3,4]. In humans, PUFAs are synthesized by fatty acid desaturases (FADSs), which are encoded by three genes on the human chromosome 11 [5] and are regulated by PUFA consumption after ingestion of linoleic acid (LA, 18:2*n*-6) and α-linolenic acid (ALA, 18:3*n*-3), which are dietary essential fatty acids in humans. However, only a small proportion of fatty acids is converted to PUFAs consisting of more than 20 carbons [6].

Since the development of cloning techniques, many desaturase genes from different species have been reported and cloned. Although eicosapentaenoic acid (EPA, 20:5*n*-3)- and DHA-producing bacteria have not been completely characterized, DNA fragments of EPA- and DHA-producing bacteria have been reported [7,8] and lower eukaryotes were reported to contain omega-3 desaturases [9]. Initially, a transgenic plant containing cyanobacterial delta-6 desaturase was developed [10], and then an omega-3 desaturase from *Caenorhabditis elegans* was cloned into mice, creating the first transgenic animals [11]. Recent biotechnological advances have enabled the production of nutri-functional fatty acids such as EPA and DHA in diverse transgenic animals and plants as well as microorganisms and have also enabled the use of conventional methods for generating these molecules from marine sources [12,13,14].

In this review, we introduce the current knowledge regarding PUFAs, their importance, and the human desaturases affecting human health and development. In addition, the well-known roles of a variety of desaturases in PUFA synthesis and recent biotechnological approaches using desaturases as transgenes for producing PUFAs are discussed.

## 2. Important Physiological Functions of PUFA

Fatty acids are important for human health and nutrition and show large compositional differences. Particularly, PUFAs containing more than two double bonds are considered to be important bioactive nutrients that regulate many physiological conditions [15]. Omega-3 and omega-6 fatty acids are major PUFAs that are named based on the position of the first double bond from the methyl end in a fatty acid chain. Omega-3 fatty acids with the first double bond on the third carbon include ALA, EPA, and DHA. Omega-6 fatty acids with the first double bond on the sixth carbon include LA, gamma-linolenic-acid (GLA, 18:3*n*-6), dihomo-gamma-linolenic acid (DGLA, 20:3*n*-6), and arachidonic acid (ARA, 20:4*n*-6). LA and ALA are typically found in plant-derived oils from corn, safflower, sunflower, canola, olive, and walnut [16]. Both LA and ALA are referred to as dietary essential fatty acids because humans cannot synthesize these molecules. However, humans are able to convert only a small portion of fatty acids to more than 20-carbon PUFAs; the conversion rates of 18-carbon fatty acids to EPA and from ALA to DHA were reported to be 5%–10% and less than 1%, respectively [17]. Therefore, fish and their oils, which contain high levels of more than 20-carbon PUFAs such as EPA and DHA, must be consumed in the diet.

PUFAs, the major structural components of cell membranes, are considered to be pivotal nutrients for preventing non-alcoholic fatty liver disease [18], autoimmune responses [19], and other chronic diseases such as cardiovascular disease [20], cancers [21], and diabetes [22]. These fatty acids, including DGLA, ARA, EPA, and DHA, provide a variety of biological effects through their capability to change the cellular membrane composition and to regulate transcription and cellular signaling [23]. Particularly, supplementation of EPA and DHA reduced inflammatory bowel disease and asthma [4]. Moreover, treatments using these fatty acids were shown to prevent colon [24] and breast cancers [25]. DHA is known to be important for cognition and brain development as well as the prevention of neurodegeneration [2]. Furthermore, DHA, a critical component in the retina, is associated with visual function in the eyes [26].

PUFAs are precursors that are metabolized to diverse lipid mediators such as eicosanoids and docosanoids, which are synthesized by cyclooxygenases or lipoxygenases [23,27]. Twenty-carbon PUFAs are known to be precursors of eicosanoids such as prostaglandins, prostacyclins, and leukotrienes, which have been linked to inflammatory and immune responses [3]. ARA is a precursor of pro-inflammatory eicosanoids, whereas EPA and DGLA are precursors of anti-inflammatory eicosanoids [3,4]. DHA is also a precursor of anti-inflammatory and immunoregulatory docosanoids such as resolvins and protectins [28]. Resolvin E and resolvin D, derived from EPA and DHA, respectively, have potent efficacy as anti-inflammatory lipid mediators in cancer prevention and treatment [29].

## 3. FADS and PUFAs Regulation in Humans

*FADS* genes code for enzymes critical for the synthesis and regulation of PUFAs. Their properties have been explored in numerous studies involving state-of-the-art techniques. Human chromosome 11q12.2-13.1 contains three *FADS* including *FADS1*, *FADS2*, and *FADS3* [30]. FADS1 is known as delta-5 desaturase and FADS2 is generally known as a delta-6 desaturase. Novel functions of FADS2, which acts on palmitic acid (16:0) and 20-carbon fatty acids for delta-8 and delta-4 desaturations, have been previously determined [31,32,33]. The three *FADS* are composed of 12 exons and 11 introns, and the classical transcripts of *FADS1* and *FADS2* encode 444 amino acids from 1335 bp, whereas *FADS3* encodes a predicted 445 amino acids from 1338 bp [5]. These molecules commonly contain an N-terminal cytochrome b5 domain and three histidine catalytic boxes, unlike stearoyl-CoA desaturases which catalyze the production of monounsaturated fatty acids such as 16:1*n*-7 or 18:1*n*-9 [30,34].

Alternative transcripts (AT) of *FADS1*, *FADS2*, and *FADS3* have been reported to be expressed in different baboon tissues and human neuroblastoma, SK-N-SH [35,36,37]. They have specific structural characteristics; for example, *FADS2AT1* includes only three histidine boxes except in the cytochrome b5 domain, suggesting that *FADS2AT1* is related to non-methylene interrupted desaturation [36]. *FADS3AT1* and *FADS3AT3* contain whole conserved structural domains of cytochrome b5 and three histidine boxes, whereas *FADS3AT5* retains intron 5 and other *FADS3AT*s have different structures. *FADS3AT*s are expressed in a tissue-specific manner and their expression is regulated according to neuronal cellular differentiation [35]. In addition, the alternative transcripts of *FADS1AT1* were identified; FADS1AT1 modulated the delta-6 and delta-8 desaturation functions of FADS2, which was the first report of the function of *FADSAT* and of the one gene’s splice variant role to regulate the other genes [37]. Unlike FADS1 and FADS2, the function of FADS3 in omega fatty acid synthesis and regulation has not been reported. However, FADS3 may catalyze an unexpected Δ13-desaturation of trans-vaccenate [38].

The diverse roles of FADS impacting human health have been evaluated using genetic and genomics approaches. Activation of fatty acid desaturation leads to pro-inflammatory conditions such as coronary artery diseases among the population consuming excessive meat and few vegetables, such as in the westernized diet [39]. A genome-wide association study showed that a single-nucleotide polymorphism (SNP) near *FADS1* was significantly associated with the plasma concentrations of EDA (eicosadienoic acid, 20:2*n*-6), EPA, and ARA [40]. *FADS* and breastfeeding are associated with differences in the IQs of children [41], and *FADS1* and *FADS2* clusters in human chromosome 11 alter the fatty acid composition in pregnant and lactating women [42]. An SNP in *FADS2* was found to be significantly associated with the occurrence of attention-deficit/hyperactivity disorder, and dietary consumption of essential fatty acids was found to be related to the dopamine pathway in attention-deficit/hyperactivity disorder in patients [43]. Microarray analysis of insulin-resistant and insulin-sensitive individuals showed that *FADS1* was differentially regulated in both adipose and muscle of insulin-resistant individuals [44], possibly through an association between neighboring SNPs and fasting glucose homeostasis [45]. Homeostasis model assessment of insulin resistance was associated with the *FADS* gene cluster as well as with fatty acid composition in serum phospholipids [44,46]; moreover SNPs mapped to the *FADS* locus were strongly associated with ARA, EPA, and DGLA in European-Americans [47]. *FADS3* was shown to be critical in hyperlipidemia [48] and implantation sites [49].

## 4. Synthetic Pathways for the Production of PUFA

**Figure 1:**
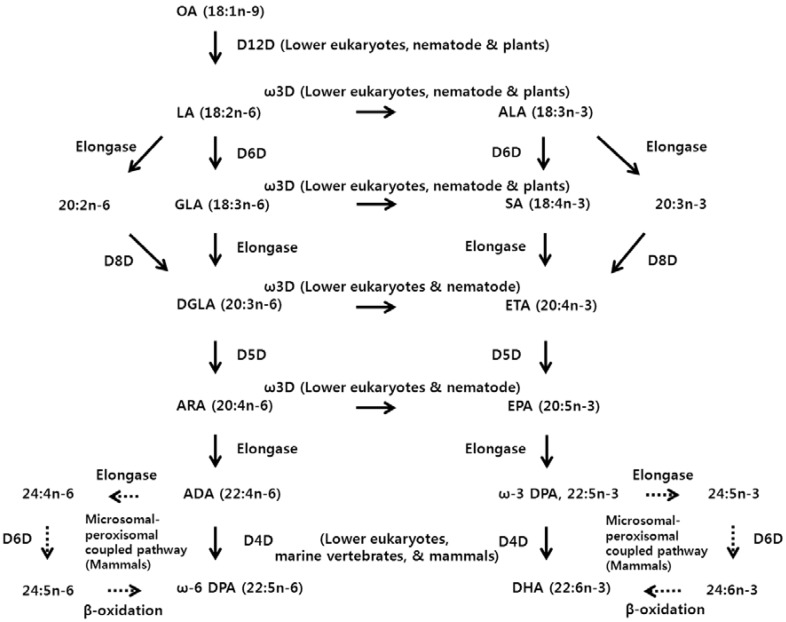
PUFA synthetic pathway to 22-carbon fatty acids from oleic acid in eukaryotic systems.

Figure 1 shows the PUFA synthetic pathways to 22-carbon fatty acids from oleic acid in eukaryotic systems via diverse fatty acid desaturases. Delta-12 desaturase and delta-15 desaturase have been identified in lower eukaryotes [50,51], plants [52,53], and animals [54,55,56,57] except mammals. These species can produce the omega-3 and omega-6 fatty acids. However, mammals, including humans, cannot synthesize omega fatty acids *de novo* and must consume them in a diet or in other nutritional supplements. Therefore, LA and ALA are known as dietary essential fatty acids for humans. Generally, after the synthesis of 18-carbon saturated fatty acid in cells, stearic acid (SA, 18:0) is desaturated to oleic acid (OA, 18:1*n*-9) by stearoyl-CoA desaturases [30]. LA is synthesized by delta-12 desaturase from OA and converted into ALA by delta-15 desaturase [58]. However, palmitic acid (PA, 16:0) is converted to palmitoleic acid (16:1*n*-7) by stearoyl-CoA desaturases [30] or into 16:1*n*-10 fatty acid by FADS2 as well [59].

Omega-3 and omega-6 fatty acids are competitive toward desaturases and elongases for the addition of carbons or double bonds in their chains, respectively. LA and ALA are converted to GLA and stearidonic acid (SA, 18:4*n*-3), respectively, by delta-6 desaturase. By two-carbon elongation, GLA and SA are metabolized to DGLA and eicosatetraenoic acid (ETA, 20:4*n*-3), respectively. These fatty acids are changed to ARA and EPA, respectively. ARA and EPA are elongated to adrenic acid (ADA, 22:4*n*-6) and omega-3 docosapentaenoic acid (ω-3 DPA, 22:5*n*-3), respectively. In mammals, it has been thought that ADA and ω-3 DPA are elongated to omega-6 tetracosatetraenoic acid (24:4*n*-6) and omega-3 tetracosapentaenoic acid (24:5*n*-3) and that delta-6 desaturated to omega-6 tetracosapentaenoic acid (24:5*n*-6), and omega-3 tetracosahexaenoic acid (24:5*n*-6) and then beta-oxidized to produce omega-6 docosapentaenoic acid (ω-6 DPA, 22:5*n*-6) and DHA (22:6*n*-3) in peroxisomes [60]. However, ω-6 DPA and DHA were found to be generated by delta-4 desaturation in primates [33], marine vertebrate [61], and lower eukaryotes [62,63]. Moreover, omega-3 desaturases, which convert omega-6 fatty acids to omega-3 fatty acids, were identified in cyanobacteria [64], some plants [52,53], lower eukaryotes [50,51], and animals such as nematodes [56].

## 5. Fatty Acid Desaturases for Synthesis of PUFA

Desaturases catalyze the introduction of double bonds between the carboxylic end of a molecule and a preexisting double bond to introduce further unsaturation into existing PUFAs or make PUFAs *de novo* in mammals deprived of dietary PUFA. These animals contain two types of desaturases to produce PUFA: front-end desaturases and methyl-end desaturases [65]. Front-end desaturases such as delta-4, delta-5, and delta-6 desaturases help to introduce double bonds between the carboxylic end of a molecule and a pre-existing double bond to generate PUFA. Based on regioselectivity, the positions of double bonds in fatty acids are referred to as delta- or omega-; desaturases are also named in this manner. The front-end desaturase was first identified in cyanobacteria [66], and subsequently identified in diverse species including plants, animals, algae, and fungi. Unlike front-end desaturases, methyl-end desaturases such as delta-12 and delta-15 desaturases (ω3 desaturases) assist in adding a double bond between pre-existing double bond and methyl end in fatty acids [30]. However, mammals, including humans, do not contain these methyl-end desaturases for producing essential fatty acids. Therefore, mammals must acquire essential fatty acids such as LA and ALA from foods or nutritional supplements.

Delta-6 desaturases catalyze the addition of a double bond at the 6th carbon-carbon bond position from the carboxylic acid end in fatty acids. Generally, delta-6 desaturases have common structural features of the N-terminal cytochrome b5-domain and histidine motifs, whereas the desaturase from *Synechocystis* sp. does not include the cytochrome b5 domain region [65]. Delta-6 desaturase is a rate-limiting enzyme in the synthesis of PUFA for generating GLA and SA from LA and ALA in mammals and humans, respectively. In addition to delta-6 desaturation activity, these enzymes showed multiple desaturase activities in mammals to produce 16:1*n*-10 fatty acid from a palmitic acid (16:0) [32] and to generate DGLA and ETA from eicosadienoic acid (EDA, 20:2*n*-6) and eicosatrienoic acid (ETE, 20:3*n*-3), respectively, as a delta-8 desaturase [31].

Delta-5 desaturases add a double bond at the 5th carbon-carbon bond from the carboxylic acid end in fatty acids. These desaturases were first identified in a fungus, *Mortierella alpina* [67,68], and subsequently have been found in many organisms. The desaturases in marine invertebrates showed bifunctional activities with delta-5 and delta-6 desaturation [69,70]. Delta-5 desaturase generally synthesizes ARA and EPA using DGLA and ETA as substrates. However, some delta-5 desaturases produce non-methylene-interrupted fatty acids such as 18:3-5, 9, and 12 and 20:4-5, 11, 14, and 17 [71,72]. Similarly, delta-5 desaturase, coded for by the FADS1 gene in primates, showed desaturation activities on EDA and ETE in breast cancer cells, suggesting that delta-5 desaturase may play a role in replacing delta-6 desaturase in a delta-6 desaturation-deficient system [73].

Delta-4 desaturases catalyze the addition of a double bond at the 4th carbon-carbon bond from the carboxylic acid end in fatty acids. Delta-4 desaturase was first identified in a lower eukaryote, *Thraustochyrium* sp. [62], and subsequently in microalgae [63], followed by the marine vertebrate delta-4 desaturase [61]. FADS2, known as a delta-6 and delta-8 desaturase, has delta-4 desaturase activity to produce ω-6 DPA and DHA from the substrates ADA and ω-3 DPA, respectively, in human cells [33]. FADS2 may be used to produce DHA in marine products using transgenic approaches.

Mammals, including humans, cannot produce omega-3 and omega-6 fatty acids because they lack delta-12 and delta-15 (ω3) desaturases. Delta-12 and delta-15 desaturase are important enzymes in the synthesis of omega-6 and omega-3 fatty acids because they add a double bond at the 12th and 15th carbon-carbon position in fatty acids, respectively, indicating that double bonds are placed at the 3rd and 6th carbon-carbon bonds from the methyl ends of fatty acids. Phylogenic analysis suggested that delta-12 desaturase is an ancestor gene of omega-3 desaturase [58]. Prokaryotic ω3 desaturases from *Synechocystis* sp., a cyanobacterium, were the first to be cloned [64], and the ω3 desaturases from *Saccharomyces kluyveri* were the first eukaryotic ω3 desaturases to be detected [51]. The delta-12 desaturase gene, *FAD2*, was first identified in *Arabidopsis thaliana*, a plant [52], and another delta-12 desaturase was found in a fungus, *Mortierella* sp. [50]. In addition to lower eukaryotes, most plants and some animals such as cockroaches and house crickets [54,55] were reported to contain delta-12 and/or delta-15 desaturases. *Caenorhabditis elegans*, a worm, contains the *fat-1* and *fat-2* genes, which catalyze the synthesis of omega-3 and omega-6 fatty acids in its physiological system [56,57,74]. These desaturase genes of *C. elegans* were first reported [56] to have the three characteristic histidine motifs without the N-terminal cytochrome b_5_ domain, unlike other plant delta-12 desaturases [58]. *fat-1* encodes a delta-12 desaturase, which has activity towards 16- and 18-carbon fatty acids, while *fat-2* is a delta-15 (ω3) desaturase that acts on 18- and 20-carbon fatty acids as well as delta-12 desaturase [57]. Moreover, delta-12 desaturation activity from *Periplaneta americana* [55] and *Acheta domesticus* [54] have been reported; however, there have been no reports of the associated genes. Importantly, ω3 desaturases have been applied to convert omega-6 fatty acids to omega-3 fatty acids to control the ratio of these fatty acids [11,12]. Omega-3 desaturases show different substrate preferences depending on the number of carbons in a fatty acid chain; ω3 desaturase from *S. kluyveri* [75] and *C. elegans* favored 18-carbon fatty acids rather than 20-carbon fatty acids [76], while ω3 desaturase from *Pichia pastoris* showed equivalent conversion rates towards 20-carbon and 18-carbon fatty acids [77]. Omega-3 desaturases from *Saprolegnia diclina* only had activity on 20-carbon fatty acids while showing a preference towards ARA [78].

## 6. PUFA Regulation and Biotechnology

Table 1 shows the PUFA biotechnology and desaturases from various organisms in nature. Although there DHA/EPA-producing bacteria have been reported, the functions of the associated genes remain unclear. Yazawa reported that *Escherichia coli* contained a genomic DNA fragment from *Shewanella putrefaciens*, which was isolated from the intestinal contents of the Pacific mackerel producing EPA [8]. *Vibrio marinus* is known to produce DHA; Morita *et al.* cloned a gene cluster from *V. marinus* MP-1 and found that it was highly homologous to DHA production-associated biosynthesis genes [7]. However, lower eukaryotes contain diverse desaturases to produce PUFAs; a delta-4 desaturase from *Isochrysis galbana* [79], delta-5 desaturase [80], and delta-12 desaturases [81] from *Thraustochytrids aureum* have been reported. A fungus, *Mortierella* sp., produced PUFAs such as ARA and EPA and the fungus contained the associated genes [50,82]. Mutants derived from *M. alpina* accumulated large amounts of ARA [83]. Furthermore, *M. alpina,* into which the ω-3 desaturase was cloned, showed higher levels of EPA than ARA [84]. Delta-12 and delta-15 desaturases from *Hansenula polymorpha* have been identified and characterized in *Saccharomyces cerevisiae* [85].

**Table 1:** PUFA biotechnology and desaturases of different organisms.

Biotechnology	Species		Associated Desaturases	References
PUFA production	Microbes & lower eukaryotes	*E. coli*	*S. putrefaciens*	[8]
		*V. marinus*	N/A	[7]
		*S. cerevisiae*	*I. galbana* D4D	[79]
			*T. aureum* D5D	[80]
			*Mortierella* D5D	[50]
			*H. polymorpha* D12D & D15D	[85]
		*T. aureum*	D12D	[81]
		*Mortierella* sp.	ω3D	[83]
			ω3D	[84]
	Plants	Tobacco	Cyanobacterial D6D	[86]
			Borage D6D	[10]
		*Brassica juncea*	*Pythium irregulare* D6D	[87]
		*A. thaliana*	*I. galbana* D9D	[88]
			*Euglena gracilis* D8D	
			*M. alpina* D5D	
		Linseed	D6D & D5D	[89]
		Soybean	*F. moniliforme* D12D/ω3D	[90]
		*A. thaliana*	*O. tauri* D6D	[14]
			*Thraustochytrium* sp. D5D	
		Linseed	*P. vialii* D6D	[91]
		Safflower seed	*M. alpina* D6D & D12D	[92]
		Rice seed	ω3D	[13]
	Animals	Mouse	*C. elegans* ω3D (*fat-1*)	[11]
		Pig	*C. elegans* ω3D (*fat-1*)	[12]
Medical application	Mammalian systems	Mouse	Fads2 (D6D)	[93]
		Mouse	Fads1 (D5D)	[94]
		Breast cancer cells	FADS1 (D5D) & FADS2 (D6D)	[73]
		Human lymphocyte	FADS1 (D5D) & FADS2 (D6D)	[95]
		Rat cortical neuron	*C. elegans* ω3D (*fat-1*)	[96]
		Mice colon cancer	*C. elegans* ω3D (*fat-1*)	[97]
		Mouse prostate cancer	*C. elegans* ω3D (*fat-1*)	[98]

N/A: not applicable; D4D: delta-4 desaturase; D5D: delta-5 desaturase; D6D: delta-6 desaturase; D8D: delta-8 desaturase; D9D: delta-9 desaturase; D12D: delta-12 desaturase; D15D: delta-15 desaturase; ω3D: omega-3 desaturase.

Higher plants generally cannot synthesize PUFAs containing more than 20-carbons, except a few species that produce GLA and stearidonic acid (SDA, 18:4*n*-3) [6]. However, because of environmental concerns regarding the use of fish oils, transgenic plant oil production has been developed even though PUFA production in transgenic species has not been completely accomplished because of metabolic bottlenecks in the complex pathway associated with a variety of genes [89,99]. In 1996, tobacco was the first transgenic plant reported; it used cyanobacterial delta-6 desaturase to accumulate GLA [86]. Borage delta-6 desaturase transgenic tobacco produced a high level of delta-6 desaturated fatty acids [10], and transgenic *B. juncea* yielded GLA using delta-6 desaturase from *P. irregulare* [87]. In 2004, different desaturases from different species, delta-9 desaturase from *I. galbana*, delta-8 desaturase from *E. gracilis*, and delta-5 desaturase from *M. alpina*, were cloned into *A. thaliana* to produce PUFAs [88]. Additional genes as well as ω3 desaturase have been cloned into plants [14,89,90]. Moreover, seed-specific expression of desaturases in tobacco and linseed as well as soybean seed was reported to produce seeds that accumulate PUFAs [6]. Recent reports showed that high levels of PUFAs not only accumulate in plant seeds [91,92], but also that ω-3 desaturase was cloned into rice seed to produce high levels of ALA [13].

Except for nematodes, animals including humans have not been reported to contain ω3 desaturase genes to change the ratio of omega-3 and omega-6 fatty acids. Transgenic animals were first engineered by introducing *fat-1* from *C. elegans* into mice; these animals produced higher levels of ω-3 fatty acids than ω-6 fatty acids in their tissues and organs [11]. Transgenic pigs containing cloned *hfat-1*, a humanized *fat-1*, were generated to produce high levels of ω-3 fatty acids, increasing the levels of these fatty acids from approximately 2% to approximately 8% [12]. It may be nutritionally beneficial to produce pork rich in ω-3 fatty acids without altering the quality of pork.

Moreover, transgenic systems using desaturases have been studied for clinical treatments or disease and physiological problem recovery. FADS2 knock-out mice with PUFA deficiency were found to have uncommon conditions involving reproduction, the skin, and the intestine [93]. FADS1 knock-out mice lacking ARA were also generated [94]. These mice could not survive for more than 12 weeks; however, supplementation with ARA helped to extend their life spans. Recently, a report showed that *FADS1* compensated for the functions of FADS2 on twenty-carbon fatty acids under FADS2-deficient conditions such as MCF-7 breast cancer cell conditions [73]. *FADS1* expression was found to be regulated by *FADS2* polymorphisms, in which insertion-deletions were observed to result from differences in major and minor alleles. Additionally, different sensitivity toward simvastatin, a lipid-lowering drug, as well as GW3965, a LXR agonist, was observed [95]. In addition to PUFA regulation of human physiological systems associated with FADS genes, *fat-1* gene transfer prohibited neuronal apoptosis in rat cortical neurons [96]. Colitis-associated colon cancer and prostate cancer were reduced in transgenic *fat-1* mice [97,98]. These results suggest that the ratio of omega-3 and omega-6 fatty acids is very important for treating neuronal function and cancer development. Thus, gene therapy using desaturases may help in the treatment or delay of many diseases as well as cancers.

## 7. Conclusions

PUFAs are important nutrient and structural components in cell membranes and are associated with human health and development. PUFAs containing 20 or more carbons are also precursors of eicosanoids or docosanoids, which are signaling molecules critical in the regulation of inflammatory and immune responses. Once humans consume LA and ALA, which are dietary essential fatty acids, diverse PUFAs are synthesized by FADS, which are known to be related to many diseases and physiological conditions. However, humans must acquire PUFAs via foods or nutritional supplements because they do not contain delta-12 desaturases and convert molecules to PUFAs very slowly. Biotechnology methods have been used to clone and introduce numerous desaturases into diverse species such as animals, plants, and microorganisms to yield the critical PUFAs EPA and DHA. In conclusion, understanding the function and regulation of PUFAs linked to human health as well as recent PUFA biotechnology techniques may facilitate the engineering of more efficient PUFAs and aid in advanced PUFA production in new transgenic species. Novel therapeutic techniques may be developed for the clinical treatment of associated diseases. Moreover, these methods may also assist in obtaining new insights into the metabolic complexity of PUFAs regulated by fatty acid desaturases.
